# Comparison of Predictive Ability of Computed Tomography and Magnetic Resonance Imaging in Patients with Carotid Atherosclerosis Complicated with Stroke

**Published:** 2019-06

**Authors:** Jianfeng ZHOU, Hongling CHEN, Tao YANG, Cuihong XING, Fengxia JIA

**Affiliations:** 1. Department of Imaging, Linyi Central Hospital, Linyi 276400, P.R. China; 2. Department of Endocrinology, Linyi Central Hospital, Linyi 276400, P.R. China; 3. Department of Medical Ward, The People’s Hospital of Zhangqiu Area, Jinan 250200, P.R. China; 4. Department of Neurosurgery, Juxian People’s Hospital, Rizhao 276599, P.R. China

**Keywords:** Carotid atherosclerosis, Stroke, Computed tomography, China

## Abstract

**Background:**

To investigate the characterizations of CT (computed tomography) and MRI (magnetic resonance imaging) in patients with carotid atherosclerosis.

**Methods:**

A retrospective analysis was performed on the medical records of 264 patients with carotid atherosclerosis underwent CT and MRI in Linyi Central Hospital, Linyi, China from January 2010 to January 2016. Among them, 142 patients with ischemic stroke were in experimental group (test group), another 122 patients in control group. The lumen stenosis degree, plaque fibrous cap status, calcification information and vascular plaque hemorrhage in the carotid artery fork of patients detected by CT and MRI were collected.

**Results:**

The detection rate of the plaque calcification of patients detected by MRI was lower than that detected by CT in the experimental group (*P*<0.05). Patients in the experimental group had higher average vascular stenosis degree detected by CT and MRI than those in the control group (*P*<0.01). The average vascular stenosis degree of patients detected by MRI was higher than that detected by CT in the experimental group (*P*<0.05). Patients in the experimental group had higher unstable fibrous cap number detected by CT and MRI than those in the control group (*P*<0.01). Patients in the experimental group had significantly higher number of vascular plaque small focus hemorrhage than those in the control group (*P*<0.05).

**Conclusion:**

Patients with carotid atherosclerotic complicated with stroke have higher plaque calcification number, vascular stenosis degree and unstable fibrous cap number. Both CT and MRI can better predict the risk of stroke.

## Introduction

Stroke has high incidence and mortality in the middle-aged and elderly population. It is the main cause of death in the elderly and loss of self-care ability in the middle-aged and elderly people in current society (1). Approximately 3/4 of survivors of stroke patients lose different degrees of labor, bringing huge burdens to society and families (2). In recent years, the incidence of stroke among young people is getting higher and higher as their lifestyles change, which poses a serious threat to human health (3). Carotid atherosclerosis is a common cerebrovascular disease. Studies (4) show that it can be used as an independent risk factor for stroke, mainly due to hypoperfusion and carotid plaque detachment as a result of the stenosis of the carotid artery lumen. In a study (5), atherosclerotic plaques were found in the intima media of stroke patients, considered that the formation and stability of plaques are closely related to stroke. Therefore, early intervention on carotid atherosclerosis is effective for preventing stroke.

Angiography has long been a gold standard for diagnosing vascular disease, but it is traumatic and expensive (6). As technology moves forward, the non-invasive detection of carotid atherosclerosis is now increasingly popular in clinical practice (7). Observing the carotid artery from different aspects and different levels, computed tomography (CT) has better practicability in displaying vascular structure and stenosis (8). Magnetic resonance imaging (MIR) is superior in analyzing the component of atherosclerotic plaques, such as hemorrhage and fibrous tissues, so as to judge the stability of plaques (9). The results of high-resolution MRI have been better consistent with those of histology (10, 11).

However, there is no relevant literature on the comparison of the predictive ability of CT and MRI in stroke. Therefore, in this study, the characterizations of CT and MRI in carotid atherosclerosis were investigated in order to provide a more efficient predictive program for the clinical prevention and treatment of stroke.

## Materials and Methods

### General information

A retrospective analysis was performed on the medical records of 264 patients with carotid atherosclerosis who underwent CT and MRI at the same time in Linyi Central Hospital, Linyi, China from January 2010 to January 2016, including 156 males and 108 females, with an average age of (65.3±6.5) yr old. Among them, 142 patients with ischemic stroke were in the experimental group, 122 patients without hemorrhagic stroke in the control group. There were no significant differences in the gender, age and BMI of patients between the two groups (Table 1).

**Table 1:** General information of case and control groups

***Factors***	***Experimental group (n=142)***	***Control group (n=122)***	χ***2***	***P***
Sex			0.014	0.907
Male	79(55.63)	67(54.92)		
Female	63(44.37)	55(45.08)		
Age (yr)			0.067	0.796
≤65	101(71.13)	85(69.67)		
>65	41(28.87)	37(30.33)		
BMI(kg/m2)			0.061	0.805
≤21	72(50.70)	60(49.18)		
>21	70(49.30)	62(50.82)		
Smoking or not			0.024	0.878
Yes	77(54.23)	65(53.28)		
No	65(45.77)	57(46.72)		
Drinking or not			0.062	0.803
Yes	79(55.63)	66(54.10)		
No	63(44.37)	56(45.90)		
Hypertension or not			0.052	0.820
Yes	95(66.90)	80(65.57)		
No	47(33.10)	42(34.43)		
Diabetes mellitus or not			0.021	0.885
Yes	92(64.79)	78(63.93)		
No	50(35.21)	44(36.07)		

Informed consent was taken from the patients before the exam and the study was approved by the local Ethics Committee of the hospital.

### Inclusion and exclusion criteria

Inclusion criteria: All patients were diagnosed with carotid atherosclerosis by angiography. Patients diagnosed with stroke by head CT or MRI were included in the experimental group. Exclusion criteria: Patients with cardiogenic cerebral embolism and neoplastic stroke were excluded; patients with MRI contraindications; patients with other severe organ diseases; patients who did not cooperate with the examination; patients with cognitive impairment and communication disorder. Willing to participate in the experiment and signing an informed consent form, all subjects and their families cooperated with the medical staff to complete relevant medical treatment.

### Experimental instruments

Lightspeed 64-row spiral CT scanner from American GE Company was used for CT. Relevant parameters were as follows: 120Kv in voltage, 220mA in electricity, 0.625mm in layer thickness and 1.15mm in pitch. The enhanced contrast agent was Ultravist 370 purchased from German Bayer Pharmaceutical Co., Ltd. A Philips Intera Archieva 3.0T superconducting magnetic resonance scanner and an 8-channel neck phased array surface coil were used for MRI.

### Experimental methods

CT: Patients were placed in a supine position, and the routine scanning ranged from the aortic arch to the vertex. After the scanning, the enhanced plain scan was performed. Then, the contrast agent was injected into the body through the cubital vein at a rate of 4 ml/s to complete the enhanced scan. Finally, vascular images were reconstructed.

MRI: Patients were placed in a supine position with the head advanced. The routine axial view 2D-TOF-MRA positioning was performed to obtain the specific position of the carotid artery fork. Then, scanning was carried out at 2 cm above and below the transverse section at the carotid artery fork perpendicular to vessels with a sequence of 3D-TOF-MRA, DIR-T1WI, FSE-T2WI and PDWI. Finally, the imaging of cervical vessels was performed. CT and MRI images were used for analyzing the plaque calcification, vascular stenosis degree, fibrous cap status and vascular plaque hemorrhage of patients with carotid atherosclerosis.

### Statistical methods

SPSS19.0 (Asia Analytics Formerly SPSS China) was used for statistical analysis. Measurement data were expressed as mean ± standard deviation, and t analysis was used for the comparison of measurement data. χ2 test was used for count data. When *P*<0.05, the difference was statistically significant.

## Results

### Comparison of plaque calcification of patients detected by CT and MRI between two groups

In the experimental group, 198 cases of plaque calcification were detected by CT in 107 patients, and 167 cases were detected by MRI in 91 patients. In the control group, 78 cases were detected by CT in 42 patients, and 55 cases were detected by MRI in 32 patients. Patients in the experimental group had higher plaque calcification number detected by CT and MRI than those in the control group (*P*<0.05). The detection rate of the plaque calcification of patients detected by MRI was lower than that detected by CT in the experimental group (*P*<0.01) (Table 2).

**Table 2:** Comparison of plaque calcification between two groups of patients (n,(%))

***Items***	***Experimental group (n=142)***	***Control group (n=122)***	***χ2***	***P***
CT	107(75.35)	42(34.43)	44.71	<0.001
MRI	91(64.08)	32(26.23)	37.70	<0.001
χ2	4.270	1.940	-	-
*P*	<0.050	0.164	-	-

### Comparison of average vascular stenosis degree of patients detected by CT and MRI between two groups

In the experimental group, the average vascular stenosis degree of patients detected by CT was (44.1±3.6) %, which detected by MRI was (55.7±3.2%). In the control group, the average vascular stenosis degree of patients detected by CT was (25.6±3.3%), which detected by MRI was (26.3±2.9%). Patients in the experimental group had higher average vascular stenosis degree detected by CT and MRI than those in the control group (*P*<0.05). The average vascular stenosis degree of patients detected by MRI was higher than that detected by CT in the experimental group (*P*<0.01) (Table 3).

**Table 3:** Comparison of average vascular stenosis degree between two groups of patients (%)

***Items***	***Experimental group (n=142)***	***Control group (n=122)***	***t***	***P***
CT	44.1±3.6	25.6±3.3	43.25	<0.001
MRI	55.7±3.2	26.3±2.9	77.70	<0.001
*t*	28.70	0.754	-	-
*P*	<0.001	0.451	-	-

### Comparison of unstable fibrous cap number of patients detected by CT and MRI between two groups

In the experimental group, the unstable fibrous cap number of patients detected by CT was 87 caps, which detected by MRI was 98 caps. In the control group, the unstable fibrous cap number of patients detected by CT was 41 caps, which detected by MRI was 49 caps. Patients in the experimental group had higher unstable fibrous cap number detected by CT and MRI than those in the control group (*P*<0.05). There was no significant difference in the unstable fibrous cap number of patients between detected by MRI and by CT in the experimental group (Table 4).

**Table 4:** Comparison of number of unstable fiber cap between two groups of patients (n,(%))

***Items***	***Experimental group (n=142)***	***Control group (n=122)***	***χ2***	***P***
CT	87(61.27)	41(33.61)	20.10	<0.001
MRI	98(69.01)	49(40.16)	22.13	<0.001
χ2	1.867	1.127	-	-
*P*	0.171	0.289	-	-

### Comparison of vascular plaque hemorrhage of patients detected by CT and MRI between two groups

CT could not detect the vascular plaque hemorrhage of patients. In the experimental group, 41 patients were detected with vascular plaque small focus hemorrhage by MRI. In the control group, 8 patients were detected. Patients in the experimental group had significantly higher number of vascular plaque small focus hemorrhage than those in the control group, with a statistically significant difference (*P*<0.05) (Table 5).

**Table 5:** Comparison of vascular intra-plaque hemorrhage between two groups of patients (n, (%))

***Items***	***Experimental group (n=142)***	***Control group (n=122)***	***χ2***	***P***
CT	0	0	-	-
MRI	41(28.87)	8(6.56)	21.62	<0.001

## Discussion

Epidemiological investigations in recent years have found that the incidence of carotid atherosclerotic plaque is increasing (12). A large number of studies (13, 14) show that carotid atherosclerosis is an independent risk factor for stroke. In a study (15), the mechanism of atherosclerosis-induced stroke was described. One is that the constant increase in atherosclerotic plaque on the vascular wall of the carotid artery causes the stenosis of the vascular wall, thereby resulting in insufficient blood supply to the brain and then stroke. The other is that the distal vascular occlusion caused by the rupture, hemorrhage and detachment of unstable plaques results in stroke. Therefore, the timely detection of carotid angiopathy is very important for the prevention of stroke. Angiography is a gold standard for carotid angiopathy. However, it is traumatic and can cause plaque detachment, which is difficult for patients with no symptom and mild symptom to accept (16). CT and MRI were increasingly valued by patients and medical staff due to their non-invasiveness, convenience and high diagnostic accuracy (17, 18). CT scan can make a better judgment on the vascular stenosis degree when it is not affected by blood flow status (19). In another study (20), CT had a sensitivity of 89% to 100% and a specificity of 94% to 100% in diagnosing the severe stenosis of vessels. The TIW1 of MRI had a high spatial resolution, and can also make a good judgment on the vascular stenosis degree. Therefore, in this paper, the characterizations of CT and MRI in carotid atherosclerosis and their predictive ability for stroke were studied. The stability of carotid atherosclerosis, closely related to the occurrence and development of stroke, is determined by the composition of the plaque (21). Therefore, CT and MRI diagnosis were performed on patients, and the image information was collected. After that, the plaque calcification, average vascular stenosis degree and unstable fibrous cap number of patients detected by CT and MRI were compared between the two groups. The results showed that patients in the experimental group had higher plaque calcification number, vascular stenosis rate and unstable fibrous cap number detected by CT and MRI than those in the control group (*P*<0.05). The findings of Zheng et al (22) are consistent with ours. It explains that carotid atherosclerosis is closely related to stroke. This is because the carotid artery is closely associated with cerebral vessels, and the calcified plaques of carotid atherosclerosis falls off into emboli, causing vascular stenosis and further stroke. Increased carotid plaque and high-risk plaques increase the risk of stroke (23).

However, the detection rate of the plaque calcification of patients detected by MRI was lower than that detected by CT in the experimental group. The average vascular stenosis degree of patients detected by MRI was higher than that detected by CT in the experimental group. There was no significant difference in the unstable fibrous cap number of patients between detected by MRI and by CT in the experimental group. This indicates that both CT and MRI can make a good judgment on the plaque calcification, vascular stenosis degree and unstable fibrous cap number, consistent with ours (24). It explains that the plaque calcification, especially small spot plaque calcification, diagnosed by CT is higher than that diagnosed by MRI. This is maybe due to the fact that calcification with different components affects and disturbs the imaging signals and the surrounding tissue signals, thereby leading to differences in the judgment of calcification. The vascular stenosis degree diagnosed by MRI was higher than that diagnosed by CT. Maybe it is because MRI can display more clearly when the vascular wall is not affected by the turbulence of the vascular stenosis. Therefore, the judgment of MRI on the vascular stenosis is more accurate. Finally, the vascular plaque hemorrhage of patients detected by CT and MRI were compared between the two groups. The results showed that CT could not detect the vascular plaque hemorrhage of patients. Patients in the experimental group had significantly higher number of vascular plaque small focus hemorrhage than those in the control group. In this study, the plaque hemorrhage showed high signal on 3D-TOF, T1WI and T2WI, but no signal was displayed in CT detection. In another study, the plaque hemorrhage was a powerful catalyst in the clinical symptoms of hemorrhage (25).

## Conclusion

Patients with carotid atherosclerotic complicated with stroke have higher plaque calcification number, vascular stenosis degree and unstable fibrous cap number than patients with carotid atherosclerosis not complicated with stroke. It can be considered that both CT and MRI can make a better judgment on the vascular condition of patients with carotid atherosclerosis, so as to predict the risk of stroke. However, in this study, the correlation between the risk factors for stroke and the carotid plaque stability and plaque composition in stroke patients was not investigated. In addition, there are relatively few studies on this at home and abroad, which cannot be cited. In the follow-up study, the experiment will be further explored.

## Ethical considerations

Ethical issues (Including plagiarism, informed consent, misconduct, data fabrication and/or falsification, double publication and/or submission, redundancy, etc.) have been completely observed by the authors.
